# Diagnosis and treatment of cervical cancer in pregnant women

**DOI:** 10.1002/cam4.2435

**Published:** 2019-08-06

**Authors:** Nitish Beharee, Zhujun Shi, Dongchen Wu, Jinhua Wang

**Affiliations:** ^1^ Department of Gynecologic Oncology Jiangsu Cancer Hospital & Jiangsu Institute of Cancer Research & The Affiliated Cancer Hospital of Nanjing Medical University Nanjing Jiangsu Province P.R. China

**Keywords:** cervical cancer, chemotherapy, diagnosis, pregnancy, pregnancy with cervical cancer, tumor management, tumor staging

## Abstract

In recent years, the incidence of gynecological malignant tumors during pregnancy has increased, mainly due to the increased number of old age pregnancy. The most common gynecological malignant tumors in pregnancy are cervical cancer, accounting for 71.6%, followed by ovarian malignant tumors, accounting for 7.0%. The incidence of cervical cancer in pregnancy is itself not very high, and the symptoms are easily confused with other diseases in pregnancy. During pregnancy, gynecological examination is limited, and therefore, the rate of misdiagnosis is higher. The treatment of cervical cancer during pregnancy is related to many factors, such as tumor size, pathological type, period of gestation, lymph node involvement, and patients' willingness to maintain pregnancy. As a reason of these factors, it is difficult to determine the optimal treatment. This article reviews the research progress on the diagnosis and treatment principles of cervical cancer in pregnancy, in order to strike a balance between effective treatment of tumors and protection of fetal health, and avoid delays in treatment and preterm delivery.

## Graphical abstract

Gynecological malignancies incidence is constantly increasing. Pregnancy complicated with cervical cancer, poses a major threat to both maternal and fetal health. Therefore, this article aims at reviewing the principles of diagnosis and treatment of such women.

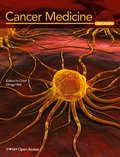

**Key message**

The effective treatment to women with pregnancy complicated with cervical cancer is mainly aimed at safeguarding both maternal and fetal health. Because of the pregnancy, it is not always easy to diagnose and treat such women, and any interventions on behalf of physicians are limited in such cases.

## 1 INTRODUCTION

Pregnancy complicated with cervical cancer refers to cervical cancer diagnosed during the current pregnancy as well as cases diagnosed 6–12 months after delivery. The incidence of pregnancy complicated with cervical cancer is low. About 1%‐3% of women diagnosed with cervical cancer are pregnant or postpartum at the time of diagnosis.^1^, ^2^ About one‐half of these cases are diagnosed prenatally, and the other half are diagnosed in the 12 months after delivery.^3^ Cervical cancer is one of the most common malignancies in pregnancy, with an estimated incidence of 0.8 to 1.5 cases per 10 000 births.^3^, ^4^, ^5^, ^6^ In China, it is reported that there are four cases of pregnancy complicated with cervical cancer per 100 000 cervical cancer patients. Multicenter data from 13 hospitals in 12 provinces in China showed that the incidence of cervical cancer during pregnancy was 0.016% (52/330 138) for the same period of gestation. Whether pregnancy can accelerate the progression of cancer is still controversial. Some scholars have found that the levels of estrogen, progesterone, and human chorionic gonadotropin during pregnancy are positively correlated with human papillomavirus (HPV) 16 and HPV 18 infection, which indirectly suggest that pregnancy may promote the progression of cervical cancer.^7^ Some studies have shown that the lymphatic circulation and blood flow of the reproductive organs of pregnant women increases, the immunity of the body decreases in the early stage of pregnancy and cervical dilation after delivery, and other factors may accelerate the metastasis of tumors, thereby accelerating the development of cervical cancer.^8^ This article reviews the progress of diagnosis and treatment of pregnancy complicated with cervical cancer.

### 1.1 Clinical manifestations

The clinical manifestations of cervical cancer in pregnant women are related to the clinical stage and diameter of the tumor. Pregnancy with early cervical cancer mostly has no obvious clinical symptoms. However, a few symptomatic patients mostly show vaginal discharge with stench, purulent or bloody secretions, and vaginal irregular bleeding. Pregnancy with late cervical cancer mainly shows pain caused by tumors or chronic anemia caused by long‐term irregular vaginal bleeding. Due to the fact that such patients are either pregnant or postpartum, the above symptoms are easily mistaken for other diseases during pregnancy or puerperium symptoms. Therefore, in pregnant patients and postpartum patients with vaginal bleeding, one should be very vigilant, and if necessary, gynecological examination and cervical exfoliation cytology screening are to be carried out.

### 1.2 Screening and diagnosis

Screening for pregnancy with cervical cancer also follows the "three‐step model", namely, cervical cytology, colposcopy, and cervical biopsy.

Cervical cytology is the first choice for rapid diagnosis of cervical cancer. The test does not pose a threat to mothers and children throughout pregnancy. Previous studies have shown that the accuracy of cervical cytological diagnosis in pregnancy is similar to that in nonpregnancy.^9^, ^10^ However, recent studies have shown that changes in maternal estrogen and progesterone levels lead to glandular hyperplasia of cervical mucosa, migration of squamous‐columnar junction, active proliferation of basal cells, irregular cell morphology, and enlargement of nuclei, which are easily misdiagnosed as highly squamous intraepithelial lesions or even invasive cancer.^7^, ^8^, ^9^ In view of the specificity of the cervix during pregnancy, it is recommended that cervical cytology smears be made by experienced pathologists who can then examine and make a conclusion on the film to reduce misdiagnosis.^10^

The cervical image under colposcopy is often difficult to identify because of the change of maternal hormone level during pregnancy. Therefore, colposcopy is better to be undertaken within the first and second trimesters of pregnancy. If the early colposcopy is not satisfactory, it can be repeated after 20 weeks of pregnancy. The indications of colposcopy include:

1. vaginal bleeding or contact bleeding excluding obstetric factors;
2. obvious abnormalities in the cervix noted during gynecological examination;
3. lesions suspicious of being an invasive cancer;
4. cervical cytology screening met the criteria of referral colposcopy^11^:
  - cervical cytology diagnosed as atypical squamous cells of undetermined significance (ASC‐US). If both ASC‐US and HPV are negative, patients with HPV‐positive can be reexamined at 6 months postpartum;
  - patients with low‐grade squamous intraepithelial lesion (LSIL); and
  - atypical squamous cells whereby high‐grade squamous intraepithelial lesion (ASC‐H) cannot be excluded; and
  - pregnant women with high‐grade squamous intraepithelial lesions (HSIL), atypical glandular cells (AGC), and above.

Cervical biopsy for suspected cervical high‐grade lesions or suspected cancers can be taken by colposcopy or naked eye for pathological examination. Cervical biopsy will not increase the incidence of complications during pregnancy, abortion rate, and premature delivery rate, but curettage of cervical canal during pregnancy will increase the abortion rate and premature delivery rate.^12^, ^13^ Therefore, this procedure is forbidden during pregnancy. In addition, the cervix during pregnancy is prone to bleeding. If the site of biopsy is too large or too deep, it can cause massive bleeding or even abortion. To minimize these risks, some scholars have proposed that the depth of biopsy should be less than 1 cm, and the biopsy should not be too large, so that bleeding can easily be stopped (if any).^7^

### 1.3 Treatment

Currently, the consensus and guidelines on the treatment of cervical cancer in pregnant women in most countries are based on small retrospective studies, and there is no unified treatment plan. Comprehensive treatment of pregnancy complicated with cervical cancer depends on many factors, such as malignant staging of tumor, gestational age, and fetal development. Multidisciplinary teams including gynecologists, oncologists, obstetricians, pathologists, and neonatal pediatricians are needed to provide patients with the best individualized treatment strategies. However, basic treatment principles should be followed, including FIGO (2009) clinical stage of cervical cancer, lymph node status, histological type of cancer, gestational age, imaging assessment (magnetic resonance imaging), and the desire for fertility of patients and families.^14^

### 1.4 Treatment of cervical intraepithelial lesions during pregnancy

According to relevant research reports, about 2/3 of cervical lesions in patients with LSIL during pregnancy will spontaneously subside, a small number of cases progress. About 1/2 of cervical lesions in patients with HSIL spontaneously subside, and patients with no progress have not been found yet,^14^ suggesting that pregnancy may rarely accelerate the progress of cervical intraepithelial lesions. Expert consensus on the management of cervical cancer in pregnancy in 2018 edition suggests that:

1. patients with cervical histology LSIL (CIN1 grade) in pregnancy can be postponed to 6 weeks postpartum for review;
2. patients with cervical histology HSIL (CIN2/3 grade) in pregnancy should be reviewed every 12 weeks after excluding invasive cervical cancer, and cervical cytology and colposcopy should be reevaluated until 6 weeks postpartum.^10^
3. If pregnancy or postpartum reexamination indicates that the disease progresses to suspicious invasive cancer, repeated biopsy should be taken.^2^
4. If highly suspected of cervical invasive cancer, cervical loop electrosurgical excision (LEEP) or cervical cold knife conization (CKC) can be performed to make a definite diagnosis rather than treatment.^15^, ^16^

The treatment of pregnancy with cervical cancer has not been well established yet, neither in China nor abroad; however, it can be treated according to the clinical stage and whether the pregnancy will be allowed to progress or will it be terminated. If pregnancy is to be terminated, the treatment is the same as that of nonpregnant women with cervical cancer. For those patients maintaining their pregnancy, specific treatment can be combined with cervical cancer staging, tumor size, gestational weeks, fetal development, and pelvic lymph node involvement.

#### 1.4.1 Pregnancy schemes for IGCS and ESGO

In 2014, the International Institute of Gynecological Oncology (IGCS) and the European Society of Gynecological Oncology (ESGO) proposed a fetal preservation treatment program.^17^

##### Treatment of stage I A1 cervical cancer 22‐25 weeks of pregnancy

Conization of cervix is an adequate and relatively safe treatment for stage I A1 cervical cancer patients.^17^ Conization of the cervix during pregnancy needs to be performed in anesthetized and well‐equipped operating rooms. The incision should not be too deep to avoid damaging the fetal membranes. A retrospective study found that women with cervical conization (>1 cm) had a higher risk of premature birth and low birth weight than women who did not undergo cervical conization.^18^ Preventive cervical cerclage can be used to prevent premature delivery and surgical bleeding.^19^

##### The treatment of stage I A2‐I B1 cervical cancer before 22‐25 weeks of gestation

1. For stage I A2‐I B1 cervical cancer, when lymph node not involved and tumor diameter less than 2 cm, if was noted that only less than 1% of the patients had parametrial extension. Therefore, conization of cervix or simple cervical resection can be carried out^17^, ^19^; or the pregnancy can be allowed to progress and treatment postponed until fetal maturity. Simple cervical resection is a less complex operation, referring to the removal of tumor 1 cm above the tumor boundary.^20^ However, radical abdominal or vaginal cervical resection during pregnancy increases the risk of early abortion. Because radical abdominal cervical resection is difficult, bleeding is high and the operation time is long (3.5 hours). Radical cervical resection is not recommended during pregnancy.^21^
2. For patients with lymph node involvement and tumor diameter less than 2 cm, termination of pregnancy is recommended, but the European Society of Clinical Oncology (ESMO) believes that neoadjuvant chemotherapy (NACT) during pregnancy and postpartum radiotherapy and chemotherapy for cervical cancer are feasible for patients with lymph node‐positive stage I B1.
3. For patients with stage I B1 cervical cancer whose tumor diameter is more than 2 cm, pelvic and abdominal lymph node resection or NACT is performed directly. If the lymph node is positive, termination of pregnancy is recommended. If the lymph node is negative or the patient has strong desire for pregnancy, NACT can be used until the fetus is mature and delivered.

##### Treatment of cervical cancer at stage I B2 and above 22‐25 weeks of gestation

According to the specific situation, NACT is the international leading method to stabilize the tumor, to prevent the progression and spread of the tumor. NACT can be used at firsthand awaiting maturity of fetus before treating the cervical cancer. For patients after 34 weeks of gestation, because of the high risk of spontaneous preterm birth, NACT is not recommended after 33 weeks of gestation.^22^

##### Treatment of cervical cancer after 22‐25 weeks of gestation

Laparoscopic pelvic lymphadenectomy is more difficult to operate with the increase of gestational weeks, so it cannot depend on the status of lymph nodes to choose treatment, however, the location of sentinel lymph nodes (SLN) in pregnancy with cervical cancer is a good indication in choosing the treatment plan. Silva et al^23^ published a case report on radioisotope SLN mapping. For a case of stage I B2 pregnancy with cervical cancer at 14 weeks of gestation, the risk of radiation to the fetus was insignificant, and the authors concluded that pelvic SLN imaging during pregnancy was feasible. However, due to the risk of allergic reactions to blue dyes used in pregnant women, the safety of this technique remains to be further studied. For stage I A2 and stage I B1 patients with tumors less than 2 cm in diameter, it is recommended to postpone treatment until fetal maturation. Early delivery or NACT is recommended when disease progression is detected. For higher grade cervical cancer, NACT is the only way to continue pregnancy and achieve fetal maturation.^17^

#### 1.4.2 The management scheme of pregnancy complicated with cervical cancer by colposcopy and cervical pathology society of China Eugenic Science Association in 2018

1. Patients in whom the pregnancy needs to be terminated, follow the principle of nonpregnant cervical cancer treatment^10^: patients with cervical cancer stages I‐IV can terminate pregnancy; for stages I B1 and below, cervical cancer patients who wants to preserve fertility functions, can undergo fertility‐preserving surgery after termination of pregnancy; but for cervical cancer patients whose pregnancy is less than 20 weeks, with a cervical cancer staging of I A2 and above, it is recommended that they undergo routine surgery for cervical cancer after termination of pregnancy.
2. The principle of individualized treatment for patients in whom the pregnancy is to be maintained.

For stage I A1 cervical cancer less than 20 weeks of gestation, pregnancy can be maintained and treatment can be initiated after delivery. Chinese scholars believe that the depth of invasion of stage I A1 cervical cancer in pregnancy is less than 3 mm and the rate of lymph node metastasis is 0.6%. It can be closely followed up by cytology and colposcopy. If there is no progress of cancer, it can be treated postpartum.^24^ Some scholars suggest that cervical conization can be used for treatment. If the pathological diagnosis of cervical conization is stage I A1 and the incision margin is positive, radical hysterectomy should be performed postpartum; if the incision margin is negative, total extrafascial hysterectomy should be performed postpartum.^11^ NACT is recommended for cervical cancer of stage I B or above at 20‐30 weeks of gestation. Because early use of NACT may easily lead to spontaneous abortion, fetal death, and fetal malformation, paclitaxel (135‐175 mg/m^2^)+cisplatin (70‐75 mg/m^2^) once every 3 weeks regimen is currently used after 20 weeks of gestation, which is relatively safe.^25^ After 2‐3 courses of chemotherapy, fetal lung maturation is promoted. However, Song et al^26^ recently reported that there was no significant difference in overall survival or progression‐free survival between pregnant women receiving cisplatin combined with other chemotherapeutic drugs and those receiving cisplatin alone. Therefore, in order to reduce adverse reactions of chemotherapy, cisplatin can be used as a single drug therapy for pregnant women with cervical cancer. Cesarean section can be performed at 35‐37 weeks of gestation to terminate pregnancy, and postpartum surgery or radiotherapy and chemotherapy can be administered to treat cervical cancer.^2^ For cervical cancer over 30 weeks of gestation, NACT is used to maintain fetal maturation. Routine one‐cycle chemotherapy and withdrawal of drugs 3 weeks before the estimated delivery time can avoid the related problems of bone marrow suppression (hemorrhage, infection, and anemia) caused by chemotherapy in mothers and infants, and avoid the accumulation of cytotoxic drugs in neonates.^17^

### 1.5 Choice of delivery mode and timing for pregnancy complicated with cervical cancer

Cesarean section is the preferred method for delivering fetuses with giant cervical tumors. Vaginal delivery carries risks of vaginal laceration, massive hemorrhage at scar incision, and metastasis of tumors. When the tumors are locally advanced, transverse cesarean section should be avoided because of the risk of cutting or tearing the tumors. Classic vertical incision can reduce bleeding and avoid damaging the blood vessels of tumors. Postoperative placenta should be sent for pathological examination to determine whether there is any metastasis. The second international consensus issued by the International Association of Gynecological Oncology in 2014 pointed out that delivery could be postponed to full‐term pregnancy (>37 weeks), but premature delivery would inevitably occur in some patients because of tumor progression or need of radiotherapy. Neonatologists should discuss the timing of delivery together at this time.

## 2 THE EFFECT OF NEOADJUVANT CHEMOTHERAPY ON FETUS AND NEWBORN

### 2.1 Influences on the fetus

The effect of chemotherapy on the fetus depends on the dose of drugs transferred to the fetus by pregnant women receiving chemotherapy during pregnancy. Calsteren et al^27^ studied placental transport of chemotherapeutic drugs commonly used in pregnant baboon models. The results showed that the average concentration of carboplatin in baboon fetal plasma was 57.5% of the maternal body; in addition, the concentration of paclitaxel in fetal umbilical cord blood was 15% of the maternal body after 3 hours of paclitaxel infusion; after transfusion of docetaxel, the concentration of docetaxel in fetal umbilical cord blood was 5%‐50% of maternal plasma, while after 26 hours, the concentration of both was high. The transplacental transmission rate of trastuzumab decreased from 85% to 3% at 2 and 26 hours after trastuzumab injection. Kohler et al^28^ suggested that there might be a platinum placental filtration mechanism because the platinum concentrations in fetal cord blood and amniotic fluid were 23%‐65% and 11%‐24% of maternal blood, respectively. Chemotherapy can directly act on the growing fetus, or indirectly act on the growing fetus through the placenta.^29^, ^30^ After the development of fetal organs, chemotherapy can affect fetal eyes, genitals, hematopoietic system, and central nervous system.^29^ Chemotherapy‐induced suppression of maternal and fetal bone marrow can also lead to anemia, which in turn affects fetal growth.^31^

### 2.2 A retrospective study on the impact of neonatal care by Song et al

It included 83 pregnant women with cervical cancer who received cisplatin‐based chemotherapy. A total of 88 neonates were born. Among them, 35 infants lost their body mass data.^26^ The average birth weight of the remaining 53 neonates was close to 2163.2 g, which was in line with the low birth weight infant standard (<2500 g). In addition, a systematic analysis of 24 studies showed that the average birth weight of newborns in pregnant women with cervical cancer treated with platinum drugs was 2213 g.^32^ Both studies have shown that NACT may lead to low birth weight.

In addition, the effect of chemotherapy on teratogenesis should not be neglected. One pregnant woman received chemotherapy with cisplatin and paclitaxel during pregnancy. The newborn was diagnosed with severe bilateral sensory hearing loss at 6 months. Another 18‐year‐old pregnant woman was treated with cisplatin and paclitaxel during pregnancy, and her baby girl suffered from retroperitoneal embryonal rhabdomyosarcoma at the age of 5. This is a rare cancer, considered to be related to genetic factors, and belongs to secondary malignant tumors associated with infant chemotherapy.^33^ Another baby girl, whose palms and feet were wrapped in a bright, tight, cellophane‐like membrane at birth, was diagnosed as ichthyosis erythema, 58 days after birth. Subsequently, the whole‐exome sequencing of newborns and their parents revealed a heterozygous neonatal mutation (c148G > A, pD50N) in the GJB2 gene. The mutation is considered to be the genetic cause of congenital ichthyosis erythema and keratitis‐ichthyosis‐deafness syndrome. However, the natural process of neonatal skin damage has not been confirmed, because this disease is rare, and erythema and skin desquamation are particularly evident in skin outbreaks induced by erythema drugs, so it cannot be denied that chemotherapeutic drugs may have the ability to aggravate fetal skin damage.

In conclusion, although the rate of low birth quality infants and fetal malformations caused by NACT during pregnancy is relatively low, the short‐term and long‐term complications of NACT for fetuses and neonates must be considered, and the possible risks during chemotherapy should be informed in detail.

## 3 CONCLUSION

The clinical manifestations of pregnancy complicated with cervical cancer are atypical, easily confused with pregnancy diseases, easily concealed by pregnancy status, and difficult to diagnose. Prenatal examinations are often neglected by pregnant women, which make it difficult to detect tumors. Therefore, conventional "three‐step" screening for cervical cancer in pregnancy is necessary. In the choice of treatment plan, we should consider both fetal and maternal factors. Conditional hospitals can set up a multidisciplinary consultation (MDT) team. Combining the clinical stages of patients, lymph node status, histological types of tumors, gestational weeks, imaging data, patients and their families' willingness to pregnancy, we can weigh the advantages and disadvantages and formulate individualized treatment plan. It is the best choice for pregnancy complicated with cervical cancer. Currently, there is no uniform standard for treatment.

## CONFLICT OF INTEREST

None declared.
